# Ventral Prostate Fibrosis in the Akita Mouse Is Associated with Macrophage and Fibrocyte Infiltration

**DOI:** 10.1155/2014/939053

**Published:** 2014-06-11

**Authors:** Sanghee Lee, Guang Yang, William Mulligan, Jerry Gipp, Wade Bushman

**Affiliations:** ^1^Department of Urology, University of Wisconsin School of Medicine and Public Health, Madison, WI 53717, USA; ^2^Cellular and Molecular Biology Program, University of Wisconsin-Madison School of Medicine and Public Health, Madison, WI 53706, USA; ^3^Carbone Cancer Center, University of Wisconsin School of Medicine and Public Health, Madison, WI 53717, USA

## Abstract

A higher incidence of lower urinary tract symptoms (LUTS) among diabetic men is unexplained. Recently, prostate inflammation and fibrosis have been implicated as major contributing factors to bladder outlet obstruction and LUTS. We characterized the inflammatory cell infiltrate and collagen content of the anterior, dorsal, and ventral lobes of 18-week-old DBA2J.Ins2-Akita mice (Akita) and age-matched control mice. We performed hematoxylin and eosin staining to score tissue injury and inflammation, picrosirius red staining to quantitate collagen content, and immunostaining to identify monocytes/macrophages and infiltrating fibrocytes. We observed significantly greater numbers of monocytes/macrophages and fibrocytes specifically in the ventral prostate of the Akita mice and found that this was associated with significant greater collagen content specifically in the ventral prostate of the Akita mice. These observations support the inference that diabetes elicits monocyte/macrophage infiltration and collagen accumulation in the prostate and suggest that further study of Akita mice may inform translational studies of diabetes in the genesis prostatic inflammation, prostatic fibrosis, and LUTS.

## 1. Introduction

Lower urinary tract symptoms (LUTS) in aging men have historically been ascribed to enlargement of the prostate gland referred to as benign prostatic hyperplasia (BPH). However, there is growing appreciation that the genesis of LUTS is likely multifactorial and, in addition to prostate enlargement, is likely to include age-related changes in bladder function, autonomic dysregulation, and metabolic influences [1]. Recently, prostate inflammation was identified as very strongly associated with severity of symptoms and prostatic fibrosis was associated both with irritative voiding symptoms such as urinary frequency and urgency and with diminished urine flow [2, 3].

Men diagnosed with symptomatic BPH have a higher incidence of diabetes than the general population [4]. Postulated mechanisms for this association include increased sympathetic tone, stimulation of prostate growth by insulin, alterations in steroid hormone secretion, inflammation, and oxidative stress. Given recent clinical studies implicating prostate inflammation and fibrosis in the genesis of LUTS, we examined the prostate of mouse model of type 1 diabetes (DBA2J.Ins2-Akita) for evidence of inflammation and/or fibrosis. This work was supported as a pilot study by the NIH Animal Model of Diabetic Complication Consortium (AMDCC).

## 2. Materials and Methods

### 2.1. Animals and Tissue Collection

Heterozygous DBA2J.Ins2-Akita (Akita) female and male mice (The Jackson Laboratory, strain number 007562, Bar Harbor, ME) were obtained for breeding. All procedures in this study were performed with 18-week-old heterozygous Akita male mice and wild type controls (WT) as approved by the Institutional Animal Care and Use Committee, University of Wisconsin-Madison. The tip of the tail from each male adult mouse were collected and sent to Transnetyx, Inc. for genotyping (Cordova, TN). At 18 weeks of age, each prostatic lobe (AP, DLP, and VP) was identified as described earlier [5] and harvested. Collected anterior prostate [6], dorsal lateral prostate (DLP), and ventral prostate (VP) were rinsed in DPBS and fixed in 10% formalin. Paraffin embedded tissues were serially sectioned (5 *μ*m). Three random areas from each prostatic lobe from each animal were acquired for H&E (Thermo Scientific, Waltham, MA, Anatech LTD, Battle Creek, MI) staining (WT: *n* = 4; diabetic: *n* = 4).

### 2.2. Characterization of Inflammatory Cells

The number and type of inflammatory cells were characterized in the prostate of Akita and WT mice. Three random areas from each prostatic lobe from each animal were acquired (WT: *n* = 4; diabetic: *n* = 4). Neutrophils were identified and quantitated in H&E stained sections. Immunohistochemistry was used to quantitate monocytes/macrophages, T lymphocytes, B lymphocytes, and fibrocytes. Briefly, sections were blocked for 4 hours in PBS containing 10% donkey serum and 1% BSA (both from Sigma-Aldrich, St. Louis, Missouri) was followed by primary antibodies-rat anti-F4/80 (1 : 50, eBioscience, San Diego, CA), rabbit anti-CD3 (1 : 100, Dako, Carpinteria, CA) and goat anti-CD20 (1 : 100, Santa Cruz, Santa Cruz, CA), and rat anti-CD45 (1 : 100, Abcam, Cambridge, MA) and rabbit anti-vimentin (1 : 100, Abcam, Cambridge, MA) overnight at 4 degrees. Secondary antibodies-donkey anti-rat Alexa 594, donkey anti-rabbit Alexa 594, donkey anti-rat Alexa 488, and donkey anti-goat Alexa 488 (1 : 100, Invitrogen, Grand Island, NY) were incubated for one hour at room temperature. Four *μ*g/mL of Hoechst 33258 (Sigma-Aldrich, St. Louis, Missouri) was incubated for 10 minutes. For quantification of each cell type, three random areas from each prostatic lobe from each animal were acquired using Nikon eclipse Ti-U microscope.

### 2.3. Collagen Quantification

Picrosirius red staining was performed with serially sectioned tissues. Paraffin embedded tissues were incubated in 0.1% sirius red solution for an hour at room temperature. Three random areas from each prostatic lobe from each animal were acquired (WT: *n* = 4; diabetic: *n* = 4) using a Spot-advanced camera on an Olympus BX51 microscope. Areas of positive staining were quantitated using MacBiophotonics Image J.

### 2.4. Statistics

Comparisons within each prostatic lobe between Akita and WT mice were performed by a two-sample *t*-test. We employed ANOVA with multiple comparisons using Fisher's protected least significant difference tests. Prior to analysis, all values were rank-transformed in order to better meet the assumptions of ANOVA. *P* values less than 0.05 were considered as significant. All analysis was performed using SAS statistical software versions 9.1 and 9.2 (SAS Institute Inc., Cary, NC).

## 3. Results

### 3.1. Increased Monocytes/Macrophages in the VP and AP of Akita Mice

**Figure 1:**
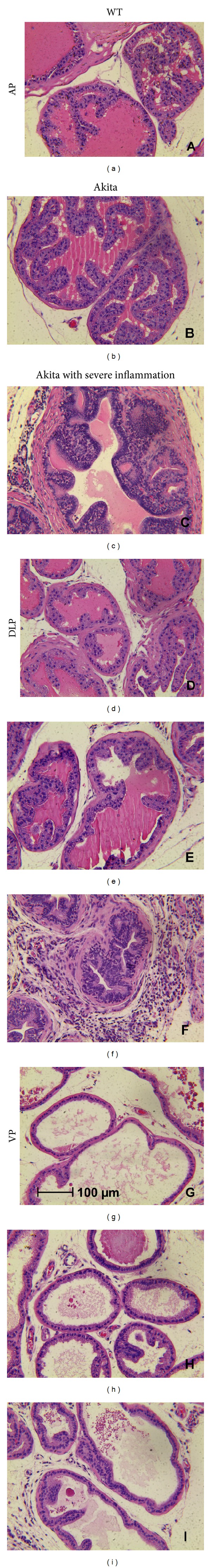
Representative H&E stained sections of the AP, DLP, and VP of 18-weeks-old DBA2J (WT: *n* = 4), DBA2J.Ins2-Aktia (diabetic: *n* = 3), or DBA2J.Ins2-Akita mice with severe inflammation (diabetic: *n* = 1).

**Figure 2:**
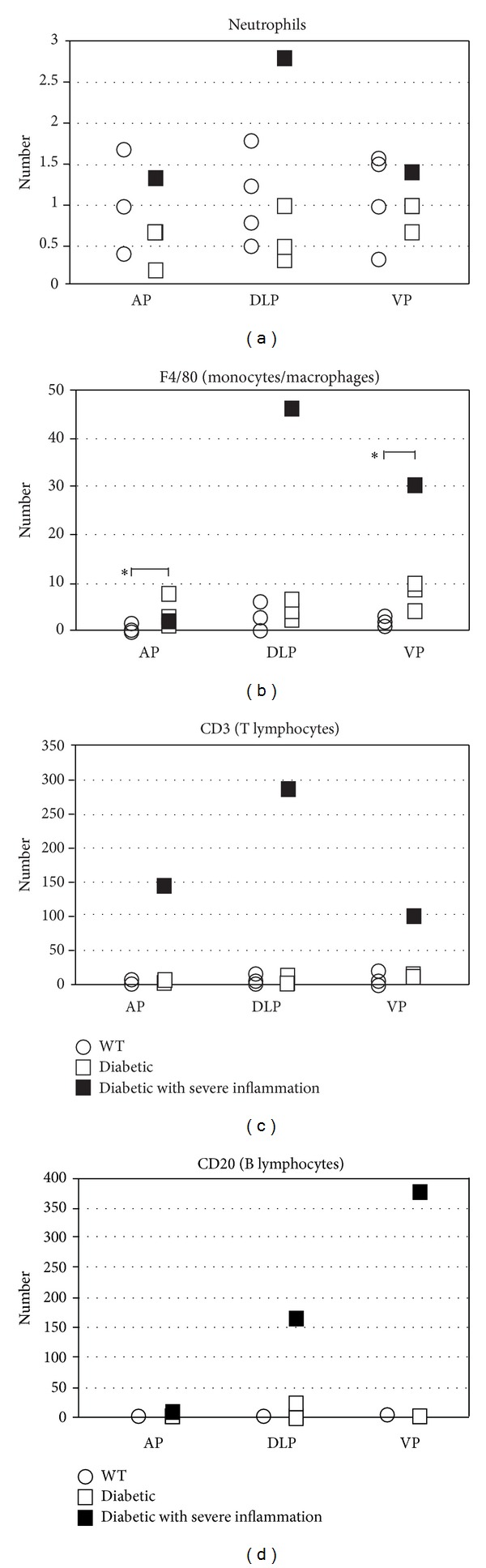
Number of inflammatory cells in the AP, DLP, and VP of 18-week-old DBA2J (WT, white circles), DBA2J.Ins2-Akita (diabetic, white squares), or DBA2J.Ins2-Akita with severe inflammation (diabetic, black squares) by H&E staining and IHC presented as the mean number of cells. (a) Neutrophils, (b) monocytes/macrophages, (c) T lymphocytes, and (d) B lymphocytes. *P value <0.05.

Prostate lobes from 18-week-old Akita and WT mice were examined for evidence of tissue injury and inflammation by routine H&E staining and scoring according to a previously published scoring system [7]. (see Figure  1 in Supplementary Material available online at http://dx.doi.org/10.1155/2014/939053). We observed no evidence of increased tissue damage, epithelial atypia or atrophy, or reactive hyperplasia. Mild inflammation was observed in all WT mice examined and three of four Akita mice (Figures 1(a), 1(b), 1(d), 1(e), 1(g), and 1(h)). The prostate from one Akita mouse exhibited severe inflammation (Figures 1(c), 1(f), and 1(i)). In order to identify any qualitative difference in the inflammatory infiltrate in WT and Akita mice, we performed immunostaining for different inflammatory cell subsets (Figure 2). For both WT and Akita mice, the infiltrate was composed primarily of T lymphocytes and monocytes/macrophages. This comparison revealed no difference in the number of neutrophils, T lymphocytes, or B lymphocytes between the WT and Akita mice. However, the number of monocytes/macrophages was significantly increased in the VP and AP of Akita mice as compared to WT. When the one Akita mouse with severe inflammation was excluded from the analysis, the number of monocytes/macrophages in the VP was still significantly increased.

### 3.2. Increased Collagen Deposition and Fibrocyte Infiltrate in the VP of Akita Mice

**Figure 3:**
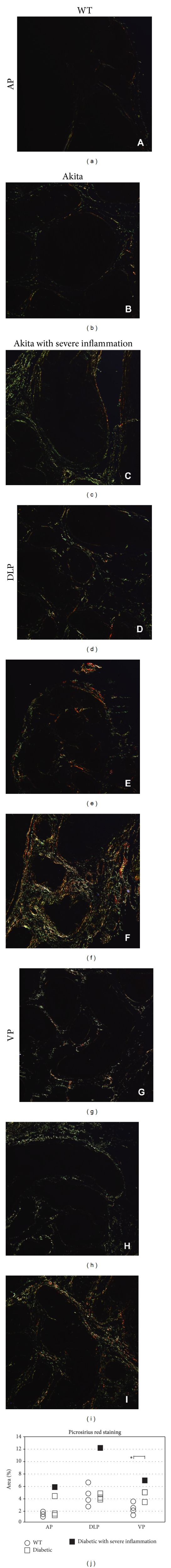
Representative section of the AP, DLP, and VP of 18-week-old DBA2J (WT; (a), (d), and (g)), DBA2J.Ins2-Akita (diabetic; (b), (e), and (h)), or DBA2J.Ins2-Akita with severe inflammation (diabetic; (c), (f), and (i)) stained for collagen with picrosirius red. Quantification results of collagen level in (a)~(i) ((j), white circles = WT, white squares = Akita, and black squares = Akita with severe inflammation). *P value <0.05.

**Figure 4:**
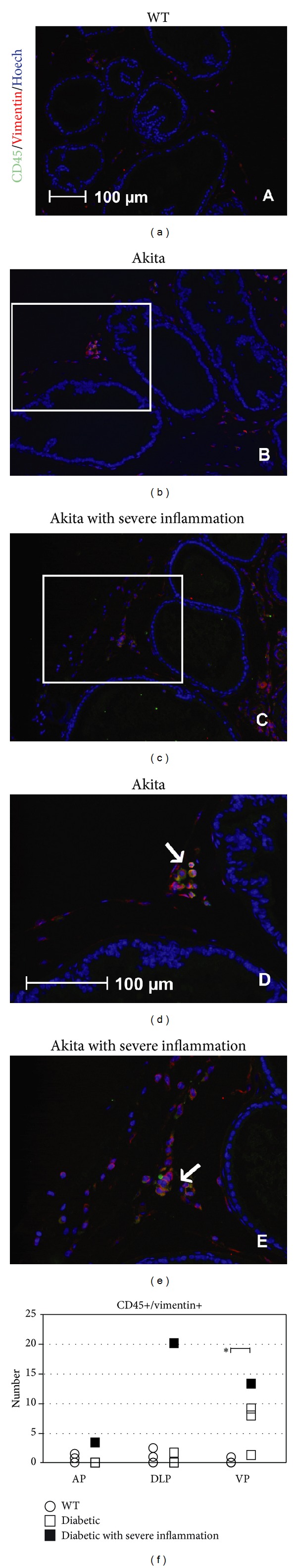
Representative section of the VP of 18-week-old DBA2J (WT; (a)), DBA2J.Ins2-Akita (diabetic; (b), (d)), or DBA2J.Ins2-Akita with severe inflammation (diabetic; (c), (e)) stained for CD45 and vimentin. Quantification results of fibrocyte infiltration in the AP, DLP (image not shown), and VP ((f), white circles = WT, white squares = Akita, and black squares = Akita with severe inflammation). *P value <0.05. Arrow denotes CD45+/vimentin+ fibrocytes.

We performed picrosirius red staining to compare collagen content in the WT and Akita mice. We observed significantly increased staining in the VP of Akita mice, a difference that remained significant even when we excluded the Akita mouse with severe inflammation (Figure 3). Fibrocytes have recently been identified as playing a key role in tissue fibrosis. These cells originate from bone-marrow derived circulating monocytes and have an intermediate phenotype of fibroblasts and macrophages. Costaining for CD45 and vimentin was performed to identify and quantitate fibrocytes in the prostate lobes of WT and Akita mice. The number of fibrocytes was significantly increased in the VP of Akita mice as compared to WT controls (Figure 4). The number of fibrocytes was still significantly increased in the VP even when we excluded the animal with severe inflammation.

## 4. Discussion

Our studies revealed increased collagen content in the VP of Akita mice as compared to age-matched controls. This conclusion is based on increased picrosirius red staining. Picrosirius red staining is considered a reliable indicator of collagen content and has been tightly correlated with tissue hydroxyproline content by a colorimetric assay [8] and HPLC (manuscript in preparation). Increased picrosirius red staining can result from increased collagen synthesis, decreased collagen degradation, or enhanced collagen cross-linking [9]. These are all changes associated with persistent wound healing—a feature of repeated injury, chronic inflammation, and prolonged cytokine release—and are cardinal aspects of fibrosis [10, 11]. The specificity of fibrosis for the VP of the Akita mouse is unexplained; however, it does echo a previous report of thickened collagen fibrils in the VP of a type 1 diabetic rat [12, 13]. Similarly, Cagnon et al. reported thickening of the extracellular matrix and reduced cell height of glandular epithelium in the VP of Streptozotocin-treated mice [14]. One of the things that confounds prostate research in rodents is the lack of correlation between the lobes of the rodent prostate and the zones of the human prostate as the anatomic and gene expression profiles for the lobes and zones are incongruent [15]. Work in various models has shown changes both in the VP [12–14] and in the DLP [16, 17]. These studies resonate with a larger body of work reporting an association of tissue inflammation and fibrosis in multiple organ systems in diabetes [6, 18–23].

It has been previously reported that tissue injury and inflammation and/or senescence results in increased collagen deposition [10, 16]. However, histologic examination and scoring of tissue damage and inflammation revealed no increase in tissue damage or generalized inflammatory infiltrate in the VP of Akita mice as compared to WT controls. However, we observed that monocyte/macrophage infiltration was significantly increased in the VP and AP of Akita mice as compared to WT controls. Monocyte/macrophage infiltration is a feature of cellular immunity that has been noted in the glomeruli of Akita mice [24] and Streptozotocin-induced diabetic mice [25] and speculated to be the source of proinflammatory cytokines that promote glomerulosclerosis [26]. Given these precedent findings, our observations might reflect a functional connection between monocyte/macrophage infiltration and VP fibrosis.

Recent studies have identified fibrocytes as having a primary role in collagen synthesis in tissue remodeling and inflammation [27]. These cells are bone marrow derived cells that take up residence in the tissue and exhibit costaining for CD45 and vimentin. Our unpublished studies of bacterial-induced prostate inflammation show robust numbers of CD45+/vimentin+ cells that costain for prolyl-4-hydroxylase, an enzyme catalyzing the formation of 4-hyroxyproline [11]. We found that CD45+/vimentin+ cells were significantly increased in the VP of Akita mice and suggest a role for fibrocytes in VP fibrosis.

The increased incidence of LUTS in diabetic men is as yet unexplained. Postulated mechanisms include accelerated rates of prostatic enlargement, bladder muscle dysfunction, and neuropathic effects on bladder function [2, 28, 29]. Given recent studies suggesting that fibrosis of the prostate produces a change in prostatic compliance that impairs opening of the bladder neck during voiding, our studies suggest that fibrosis instigated by monocyte/macrophage infiltration may be one mechanism by which diabetes contributes to development of LUTS in men. The possible effect of prostate fibrosis on opening of the bladder neck is uniquely important in humans because the encapsulated prostate completely surrounds the bladder neck and urethra. Since the prostate in mice is not encapsulated and the arrangement of the prostate and bladder neck is such that fibrosis would not be expected to impinge on either the bladder neck or urethra, we are unable to specifically evaluate the effect of the fibrosis we observe on voiding function in this model. Even so, it is a valuable model in which mechanistic studies may be performed to understand how diabetes-induced inflammation induces prostatic fibrosis.

## 5. Conclusions

These observations support the inference that diabetes elicits monocyte/macrophage/fibrocyte infiltration and collagen accumulation in the ventral prostate and suggest that further study of Akita mice may inform translational studies of diabetes in the genesis of BPH, prostatic inflammation and fibrosis, and LUTS.

## Supplementary Material

The tissue was graded in three randomly selected 20x fields of H&E stained sections according to three criteria; inflammatory infiltrate, tissue damage and hyperplasia based on the criteria shown in previous publication (reference #7). Total score in each field was calculated as intensity x focality. Inflammatory infiltrate is the only criteria showing significant difference between 18 week old Akita and WT mice in the DLP.

## Abbreviations

Akita: DBA2J.Ins2-Akita (heterozygous diabetic)
AMDCC: Animal Models of Diabetic Complications Consortium
AP: Anterior prostate
BPH: Benign prostatic hyperplasia
DLP: Dorsal lateral prostate
H&E: Hematoxylin-Eosin
LUTS: Lower urinary tract symptoms
VP: Ventral prostate
WT: Wild type.
