# Supplementary material for: Ventral Prostate Fibrosis in the Akita Mouse Is Associated with Macrophage and Fibrocyte Infiltration
Source: J Diabetes Res. 2014 Jun 11;2014:939053. doi: 10.1155/2014/939053 (PMC4074948; doi:10.1155/2014/939053)
Supplement: Supplementary file 1 — The tissue was graded in three randomly selected 20x fields of H&E stained sections according to three criteria; inflammatory infiltrate, tissue damage and hyperplasia based on the criteria shown in previous publication (reference #7). Total score in each field was calculated as intensity x focality. Inflammatory infiltrate is the only criteria showing significant difference between 18 week old Akita and WT mice in the DLP. [file 939053.f1.pdf]

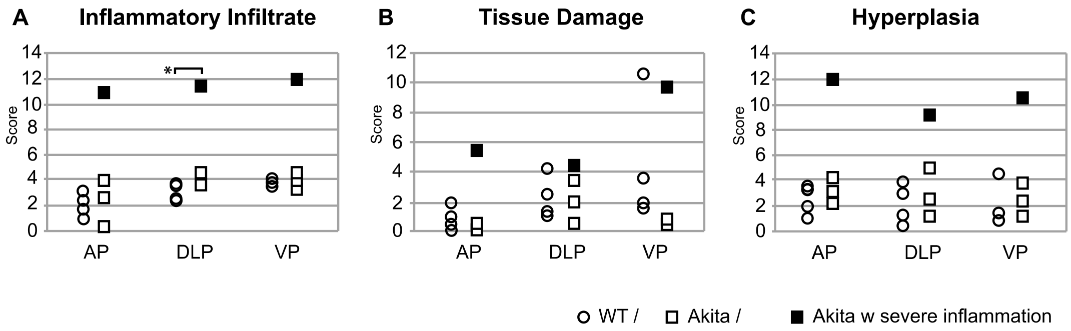

Supplementary Figure 1. Compartment of inflammatory infiltrate (A), tissue damage (B) and hyperplasia (C) between 18-week-old Akita and WT mice in each prostatic lobe; the AP, DLP and VP of 18-week-old DBAJ2 (WT, white circles), DBA2J.Ins2-Akita (diabetic, white squares) or DBA2J.Ins2-Akita with severe inflammation (diabetic, black squares). *P-value < 0.05.
